# Intralesional 5-Fluorouracil for Keloids: A Systematic Review

**DOI:** 10.1177/12034754241256346

**Published:** 2024-05-28

**Authors:** Aliyah King, Marina Guirguis, Seyyon Satkunanathan, Mysa Saad, Reetesh Bose

**Affiliations:** 1 Faculty of Medicine, University of Ottawa, Ottawa, ON, Canada; 2 Faculty of Health Sciences, University of Ottawa, Ottawa, ON, Canada; 3 Division of Dermatology, The Ottawa Hospital and University of Ottawa, Ottawa, ON, Canada

**Keywords:** 5-fluorouracil, keloids, intralesional injections, systematic review

## Abstract

Keloids are benign, fibroproliferative dermal tumours, often arising after trauma, that are more common in darker skin types. Numerous therapeutic options have been employed for the treatment of keloids; however, there is no one gold standard approach. Five-fluorouracil, a potent chemotherapeutic agent, has emerged as a promising therapeutic option. Therefore, this systematic review, using Preferred Reporting Items for Systematic Reviews and Meta-Analyses (PRISMA) guidelines, focused on providing a broad overview of the use of 5-fluorouracil for the management of keloids. Forty studies (2325 patients) met inclusion criteria and investigated 5-fluorouracil for keloid management, with 19 studies (1043 patients) including a 5-fluorouracil monotherapy group. Five-fluorouracil monotherapy demonstrated consistent keloid improvement with >254 keloids injected across various anatomical regions. Five-fluorouracil monotherapy was most often compared to intralesional triamcinolone acetonide, utilizing the Patient and Observer Scar Assessment Scale and the Vancouver Scar Scale. The most common keloid parameters assessed were height, size, volume, width, length, induration, pruritus, and erythema. Five-fluorouracil monotherapy exhibited substantial improvements, with weight averages of 73% of patients experiencing >25% improvement and 67% achieving >50% improvement. Relapse rate was 16% at 27 weeks after 5-fluorouracil monotherapy treatment. Limitations included potential selection bias, language restrictions, and heterogenous data analysis among studies. Overall, our findings underscore the potential effectiveness of 5-fluorouracil monotherapy in the management of keloids, with an encouraging safety profile. Larger prospective trials are needed to determine optimal therapy or combination therapy for the management of keloids. This detailed compilation of treatment protocols, outcomes, and relapse rates stand as a valuable resource for further research and clinical applications.

## Introduction

Keloids are benign, fibroproliferative dermal tumours that rarely regress.^1^ Onset typically follows trauma (e.g. piercing, tattooing, scratching, burns), and can be linked to genetic predisposition, environmental factors, and mechanical tension at the wound site.^1-3^ Keloids are more common in darker skin types, particularly in Black, Asian, and Hispanic individuals, and the impact of disease may be greater in certain cultures.^2,4^ There is thought to be a pathophysiologic difference between hypertrophic scarring and keloids, but this distinction is not well understood.^1^

Although the exact etiology remains to be understood, it is widely accepted that keloids result from an exaggerated wound healing response characterized by excessive collagen deposition, fibroblast proliferation, and altered extracellular matrix dynamics.^5,6^ This pathological remodelling of tissue leads to the formation of raised scars that can cause discomfort, pruritus, pain, and aesthetic concerns.^7,8^ They may also affect motor function by impeding joint movement, thereby decreasing functional performance and quality of life.^5^ Keloids often occur in conspicuous locations, such as the face, ears and neck, further exacerbating the psychosocial impact on patients.^9^

There is no universal treatment strategy for keloids; however, numerous treatment options have been reported on, including intralesional corticosteroid and other types of injectable medications, silicone sheets, laser therapy, radiotherapy, and surgical excision.^2,4,10^ Among the various treatments that have emerged, 5-fluorouracil (5-FU), a potent chemotherapeutic agent, has shown promise in the management of keloids.^9^ This systematic review aims to provide a broad overview of the use of 5-FU monotherapy as a therapeutic option for keloid management. Through a narrative-based approach, we aim to delve into the potential benefits and treatment approach of 5-FU monotherapy for keloids, providing a contextualized exploration to elucidate its efficacy, discuss combination therapies studied, and guide future therapeutic considerations.

## Methods

Our study protocol followed the Preferred Reporting Items for Systematic Reviews and Meta-Analyses (PRISMA) guidelines^11^ and is registered on PROSPERO (CRD42023457776).

### Eligibility Criteria

All English-language, original studies evaluating the efficacy of 5-FU for the management of keloids were included in the data extraction and are detailed in the Supplemental Material. To focus on the efficacy of 5-FU for the management of keloids, this review specifically concentrated on studies involving a 5-FU monotherapy group. Studies with less than 10 patients, in vitro studies, animal studies, and incomplete studies were excluded.

### Literature Search Strategy

Our literature search strategy was developed with the guidance of an information specialist (Risa Shorr, Librarian, The Ottawa Hospital). From inception to April 25, 2023, EMBASE, MEDLINE, and CENTRAL were searched using the keywords: (fluorouracil OR 5-FU OR 5FU OR Carac OR Tolak OR Efudex OR Fluoroplex) AND (keloid). No date or language restrictions were applied. Supplemental Figure S1 outlines the study selection process.

### Study Selection, Data Extraction, Evidence Synthesis, and Study Outcomes

Three independent reviewers screened the studies based on the inclusion and exclusion criteria using Covidence systematic review software. Two additional reviewers confirmed all information and resolved conflicts. Using Microsoft Excel, all reviewers extracted the following study characteristics: author information, publication date, country, study design, number of participants, sex of population, racial demographics, mean age of population, site of keloids, interventions, outcomes, attrition rates, and relapse rates. The data extraction table was used to synthesize information for the review and quantitative analysis. The primary outcomes of this narrative-based systematic review were patient characteristics, keloid location, treatment protocol, clinical outcomes, follow-up period, and relapse rates of 5-FU monotherapy groups.

### Risk of Bias Assessment

The Grading of Recommendations, Assessment, Development, and Evaluations (GRADE) scale was used to assess study risk of bias and quality of evidence. Of the 40 studies extracted and included in Supplemental Table S1, 20 were randomi-zed controlled trials [GRADE assessment = 20 (100%) high],^2,4,5,7,10,12-26^ 11 were prospective comparative studies [GRADE assessment = 11 (100%) moderate^1,6,8,9,27-33^], 7 were prospective noncomparative studies [GRADE assessment = 7 (100%) moderate^3,34-39^], 1 was a retrospective comparative study [GRADE assessment = 1 (100%) moderate^40^], and 1 was a retrospective non-comparative study [GRADE assessment = 1 (100%) low^41^]. Of the 19 studies including a 5-FU monotherapy subanalysis, 7 were randomized controlled trials,^4,10,12,19,22,23,26^, 9 were prospective comparative studies^1,6,8,9,27,29,31-33^, and 3 were prospective noncomparative studies.^34-36^

## Results

There were 458 studies that underwent title and abstract screening after using the search strategy and removing duplicates (Supplemental Figure S1). A total of 374 were found to not be relevant to our research question, and 84 studies underwent full-text screening. After full-text review, 44 studies were excluded (Supplemental Figure S1), leaving 40 studies that underwent analysis. Included studies represented 2325 patients, cumulatively [weighted mean age 30.1 (range 5-81); 57% female; Supplemental Table S2]. Two studies reported specific data on Fitzpatrick skin phototypes [Type III = 110 (50.8%); Type IV = 64 (34.8%); and Type V = 10 (5.4%)].^3,12^ Of 15 studies reporting the number and locations of keloids injected with 5-FU, >644 keloids were injected [>401 trunk (62.27%); 116 ears (18.01%); 74 head and neck (11.49%); >52 limbs (8.07%); >1 other (0.16%); Supplemental Table S3].^3,5,17,21,23,26-28,30,34-36,39,41,42^ Treatment protocols, outcomes, and relapse rates for all studies can be found in the Supplemental Material.

Nineteen studies (7 RCTs, 8 prospective comparative, and 4 prospective noncomparative) included a 5-FU monotherapy group and represented 1043 patients [weighted mean age 32.0 (range 11-81); 56% female; Supplemental Table S2]. Of 812 patients receiving 5-FU monotherapy, the 5-FU solution (50 mg/mL) injected volume ranged from 0.02 to 0.4 mL/cm (Supplemental Table S4). The average interval between 5-FU injections was 2.5 weeks (range: 1-4.2) for a total of 15.8 (range: 6-26) weeks. The average follow-up post-5-FU injections were 28 weeks (range: 6-52). Of 7 studies reporting the number and locations of keloid injections with 5-FU monotherapy, >254 keloids were injected at various sites: >191 trunk (75%); >32 limbs (13%); 16 ear (6%); 15 head and neck (6%) (Supplemental Table 5).^8,23,26,27,34-36^

Comparative studies most often had 5-FU injections and triamcinolone acetonide injections as experimental groups.^1,4,10,12,19,22,23,25-27,29,31,33^ Other comparative groups included cryotherapy,^1,10,32^ verapamil,^12^ platelet-rich plasma,^12^ bleomycin,^29^ botulinum toxin,^8^ and excision^1^ (Supplemental Table S4). The most common standardized outcome assessment scales included the Vancouver Scar Scale (2/19 studies; 11%^26,29^) and the Patient and Observer Scar Assessment Scale (1/19 studies; 5%^12^). The most common outcomes assessing keloid appearance included height (14/19 studies; 74%^1,4,8,10,19,22,23,25-27,29,33,34,36^), size (7/19 studies; 37%^1,4,10,22,23,31,36^), volume (4/19 studies; 21%^6,10,33,35^), width (3/19 studies; 16%^6,31,33^), length (2/19 studies; 11%^31,33^), and induration (2/19 studies; 11%^1,36^). The most common adverse events assessed included pruritus (8/19; 42%^1,6,8,25,26,33,35,36^) and erythema (1/19 studies; 5%^6^; Supplemental Table 6). Outside of well-known side effects (eg, pain, hyperpigmentation, atrophy), no major adverse events were reported.

All keloid outcomes assessed improved with 5-FU monotherapy (Supplemental Table 7), with 73% of patients who experienced >25% improvement, and 67% who experienced >50% improvement in keloids (Supplemental Table 8).^1,4,8,12,22,23,25,27,33-36^ One study of 40 patients found a 57% improvement in the Patient and Observer Scar Assessment Scale.^12^ In another study of 30 patients, the Vancouver Scar Scale improved by 54%.^29^ Improvement ranges for height, size, and volume of keloids were 43% to 89% (4 studies of 70 patients),^10,22,26,27^ 32% to 70% (22 studies of at least 28 patients^10,36^), and 61% (1 study of 33 patients),^10^ respectively. From 6 studies encompassing 138 patients, pruritus improved between 35% and 100%.^22,25,26,33,36,42^ Induration and erythema assessed by one study of 20 patients saw improvement rates of 83% and 78%, respectively.^22^ Of 331 patients, 53 (16%) relapsed at an average of 27 weeks (Supplemental Table S9).^8,12,19,23,25-27,29,31,33-36^

## Discussion

Keloids are common, benign fibroproliferative scarring reactions that more commonly affect individuals with richly melanated skin types, including individuals of African, Asian and Hispanic ancestry.^2,4^ Keloids commonly result from aberrant wound healing following injury to the dermis incited by trauma or inflammation.^1-3^ In addition to pain, pruritus, ulcers and restricted function, the sociocultural and psychological implications associated with keloids may cause more distress to individuals from certain ethnic backgrounds.^43,44^

The pathogenesis of keloids involves a complex interplay between inflammatory cells, including fibroblasts, mast cells, keratinocytes, melanocytes, and vascular endothelial cells.^45,46^ An exaggerated inflammatory phase with overexpression of cytokines and growth factors such as transforming growth factor beta-1 (TGF-β1)and transforming growth factor beta-2 (TGF-β2), result in increased fibroblast activity and subsequent extracellular matrix collagen formation and deposition.^47^ Other inflammatory proteins including vascular endothelial growth factor and platelet-derived growth factor are also thought to play a role in increased collagen synthesis.^46,48^ These may represent potential therapeutic targets; however, further research is required to fully elucidate the pathophysiological mechanisms underlying keloid formation.^47^

While numerous treatment options exist for keloids, treatment remains clinically challenging due to tendency to grow and the high likelihood of recurrence.^49^ Conventional therapies include occlusive dressings such as silicone sheets, which may decrease inflammation and reduce the risk of excess scar formation.^50^ Compressive therapy, including elastic wrap bandages, magnets, and custom pressure molds, may be used as adjunctive therapy to prevent recurrence such as following surgical excision.^51^ Intralesional corticosteroids (i.e. triamcinolone acetonide) are a mainstay of treatment and may work by decreasing TGF-β expression, thereby reducing fibroblast activity.^52^ Intralesional 5-FU has also been shown to be effective in reducing collagen synthesis and fibroblast proliferation.^53^ Other emerging intralesional injections include bleomycin, mitomycin C, botulinum toxin A, and platelet-rich plasma. Cryotherapy including intralesional cryotherapy may be an effective option for smaller lesions.^54^ Light-based therapies such as pulsed-dye laser and ablative laser (e.g. carbon dioxide or erbium YAG) are typically recommended as adjunctive therapies prior to surgical excision.^55^ Laser-assisted drug delivery, which enhances penetration of agents such as corticosteroids, is another therapeutic option that has recently been investigated and may potentially improve the efficacy of other topical or intralesional therapies.^56^ Finally, surgical excision followed by radiation therapy has been shown to be effective and reduces the risk of recurrence.^57^

The Patient and Observer Scar Assessment Scale considers patient and observer assessment of various scar attributes such as pain, pruritus, pigmentation, stiffness, vascularity, thickness, relief, and pliability^58^; whereas, the Vancouver Scar Scale is a clinical evaluation of vascularity, pigmentation, pliability, and height of the scars to quantify scar severity.^59^ Height, size, volume, width, length, induration, pruritus, and erythema are also commonly assessed parameters for keloids. The literature quite consistently reports 5-FU monotherapy to be effective, however, relapses did occur frequently. These findings emphasize the need for continued research into the potential efficacy for 5-FU monotherapy in the management of keloids. Our comprehensive compilation of treatment protocols, outcomes, and release rates, detailed in the Supplemental Materials, offers a valuable resource for future research and clinical applications.

There are several limitations of the present study. Our search strategy and screening process may have inadvertently failed to capture all relevant studies, resulting in selection bias. Included studies were limited to those published in the English language which would have resulted in exclusion of studies not available in English, some which may be from regions with a higher prevalence of keloids and different management practices than in North America. The studies included in this review often employed inconsistent data analysis methods as well as heterogeneity of outcome measures and results, making it challenging to make comparisons across different studies; however, high-level evidence from randomized controlled trials and comparative studies were included. The positive outcomes and safety profile observed underscores the benefit of intralesional 5-FU monotherapy as a therapeutic option for keloids. Further research and larger randomized-controlled, and prospective trials are essential in further understanding and improving the management of keloids.

## Supplemental Material

sj-docx-1-cms-10.1177_12034754241256346 – Supplemental material for Intralesional 5-Fluorouracil for Keloids: A Systematic Review Supplemental material, sj-docx-1-cms-10.1177_12034754241256346 for Intralesional 5-Fluorouracil for Keloids: A Systematic Review by Aliyah King, Marina Guirguis, Seyyon Satkunanathan, Mysa Saad and Reetesh Bose in Journal of Cutaneous Medicine and Surgery

sj-png-2-cms-10.1177_12034754241256346 – Supplemental material for Intralesional 5-Fluorouracil for Keloids: A Systematic Review Supplemental material, sj-png-2-cms-10.1177_12034754241256346 for Intralesional 5-Fluorouracil for Keloids: A Systematic Review by Aliyah King, Marina Guirguis, Seyyon Satkunanathan, Mysa Saad and Reetesh Bose in Journal of Cutaneous Medicine and Surgery
