# Supplementary material for: Intralesional 5-Fluorouracil for Keloids: A Systematic Review
Source: J Cutan Med Surg. 2024 May 28;28(4):381–6. doi: 10.1177/12034754241256346 (PMC11403916; doi:10.1177/12034754241256346)
Supplement: sj-docx-1-cms-10.1177_12034754241256346 – Supplemental material for Intralesional 5-Fluorouracil for Keloids: A Systematic Review [file sj-docx-1-cms-10.1177_12034754241256346.docx]

**Supplemental Table S1.** Complete reference list of studies with 5-fluorouracil monotherapy and non-monotherapy groups.

| **First author, publication year** | **Reference** |
| --- | --- |
| (Albalat, 2022) | Albalat, W., Nabil, S., & Khattab, F. (2022). Assessment of various intralesional injections in keloid: comparative analysis. *Journal of Dermatological Treatment*, *33*(4), 2051-2056. |
| (Ali, 2021) | Ali, N., Arif, M., Shahid, M., Tahir, K., Jawaid, K., & Hussain, A. (2021). Comparison of efficacy of intralesional 5-Flourouracil plus triamcinolone acetonide verses intralesional triamcinolone acetonide alone in the treatment of keloids. *Journal of Pakistan Association of Dermatologists*, *31*(1), 58-62. |
| (Ali, 2016) | Ali, K. A. S. H. I. F., Tayyaba, F. A. R. H. A. T. U. L. L. A. N. N., & Tabassum, H. M. (2016). Comparison between the efficacy of intra-lesional triamcenolone and combination of triamcinolone with 5-fluorouracil in the treatment of keloid and hypertrophic scars. *Pakistan J Med Health Sci*, *10*(2), 578-81. |
| (Asilian, 2012) | Asilian, A., Darougheh, A., & Shariati, F. (2006). New combination of triamcinolone, 5‐fluorouracil, and pulsed‐dye laser for treatment of keloid and hypertrophic scars. *Dermatologic Surgery*, *32*(7), 907-915. |
| (Chen, 2017) | Chen, X. E., Liu, J., Bin Jameel, A. A., Valeska, M., Zhang, J. A., Xu, Y., ... & Zhou, B. R. (2017). Combined effects of long‑pulsed neodymium-yttrium-aluminum-garnet laser, diprospan and 5-fluorouracil in the treatment of keloid scars. *Experimental and Therapeutic Medicine*, *13*(6), 3607-3612. |
| (Darougheh, 2007) | Darougheh, A., Asilian, A., & Shariati, F. (2009). Intralesional triamcinolone alone or in combination with 5‐fluorouracil for the treatment of keloid and hypertrophic scars. *Clinical and experimental dermatology*, *34*(2), 219-223. |
| (Deng, 2021) | Deng, K., Xiao, H., Liu, X., Ogawa, R., Xu, X., & Liu, Y. (2021). Strontium-90 brachytherapy following intralesional triamcinolone and 5-fluorouracil injections for keloid treatment: A randomized controlled trial. *PloS one*, *16*(3), e0248799. |
| (Hatamipour, 2011) | Hatamipour, E., Mehrabi, S., Hatamipour, M., & GHAFARIAN, S. H. R. (2011). Effects of combined intralesional 5-fluorouracil and topical silicone in prevention of keloids: a double blind randomized clinical trial study. |
| (Hietanen, 2020) | Hietanen, K. E., Järvinen, T. A., Huhtala, H., Tolonen, T. T., & Kaartinen, I. S. (2020). Histopathology and immunohistochemical analysis of 5‐fluorouracil and triamcinolone treated keloids in double‐blinded randomized controlled trial. *Wound Repair and Regeneration*, *28*(3), 385-399. |
| (Khalid, 2019) | Khalid, F. A., Mehrose, M. Y., Saleem, M., Yousaf, M. A., Mujahid, A. M., Rehman, S. U., ... & Tarar, M. N. (2019). Comparison of efficacy and safety of intralesional triamcinolone and combination of triamcinolone with 5-fluorouracil in the treatment of keloids and hypertrophic scars: Randomised control trial. *Burns*, *45*(1), 69-75. |
| (Khalid, 2018) | Khalid, F. A., Farooq, U. K., Saleem, M., Rabbani, J., Amin, M., Khan, K. U., ... & Tarar, M. N. (2018). The efficacy of excision followed by intralesional 5-fluorouracil and triamcinolone acetonide versus excision followed by radiotherapy in the treatment of ear keloids: a randomized control trial. *Burns*, *44*(6), 1489-1495. |
| (Li, 2022) | Li, Y., Zhang, D., Hang, B., & Wang, H. (2022). The efficacy of combination therapy involving excision followed by intralesional 5-fluorouracil and betamethasone, and radiotherapy in the treatment of keloids: a randomized controlled trial. *Clinical, Cosmetic and Investigational Dermatology*, 2845-2854. |
| (Manzoor, 2020) | Manzoor, H., Tahir, K., Nasir, A., Mufti, S., & Shehzad, A. (2020). Comparison of efficacy of intralesional 5-fluorouracil alone, intralesional triamcinolone acetonide alone and intralesional triamcinolone acetonide with 5-fluorouracil in management of keloids. *Journal of Pakistan Association of Dermatologists*, *30*(2), 282-285. |
| (Naseem, 2022) | Naseem, S., Paracha, M. M., Sagheer, F., Qayyum, A., & Noor, S. M. (2022). COMPARISON OF EFFICACY OF INTRALESIONAL TRIAMCINOLONE ACETONIDE VERSUS COMBINATION OF INTRALESIONAL TRIAMCINOLONE WITH 5-FLUOROURACIL IN TREATMENT OF KELOIDS: A RANDOMIZED CONTROLLED TRIAL. *Journal of Postgraduate Medical Institute*, *36*(4), 207-12. |
| (Sadeghinia, 2011) | Sadeghinia, A., & Sadeghinia, S. (2012). Comparison of the efficacy of intralesional triamcinolone acetonide and 5‐fluorouracil tattooing for the treatment of keloids. *Dermatologic surgery*, *38*(1), 104-109. |
| (Sagheer, 2016) | Sagheer, A., Shehzad, A., & Hussain, I. (2016). Comparison of efficacy of intralesional 5-fluorouracil alone versus intralesional triamcinolone acetonide with 5-fluorouracil in small keloids. *Journal of Pakistan Association of Dermatologists*, *26*(4), 361-365. |
| (Saleem, 2017) | Saleem, F., Rani, Z., Bashir, B., Altaf, F., Khurshid, K., & Pal, S. S. (2017). Comparison of efficacy of intralesional 5-fluorouracil plus triamcinolone acetonide versus intralesional triamcinolone acetonide in the treatment of keloids. *Journal of Pakistan Association of Dermatologists*, *27*(2), 114-119. |
| (Sharma, 2012) | Sharma, S., Bassi, R., & Gupta, A. (2012). Treatment of small keloids with intralesional 5-fluorouracil alone vs. intralesional triamcinolone acetonide with 5-fluorouracil. *Journal of Pakistan Association of Dermatologists*, *22*(1), 35-40. |
| (Srivastava, 2018) | Srivastava, S., Patil, A. N., Prakash, C., & Kumari, H. (2017). Comparison of intralesional triamcinolone acetonide, 5-fluorouracil, and their combination for the treatment of keloids. *Advances in Wound Care*, *6*(11), 393-400. |
| (Alamdari, 2018) | Alamdari, H. A., Davarnia, G., Ghadim, H. H., & Sadri, A. (2018). Intralesional Cryotherapy Versus Intralesional Corticosteroid and 5-Fluorouracil in the Treatment of Hypertrophic Scars and Keloids: A Clinical Trial. *Crescent Journal of Medical & Biological Sciences*, *5*(3). |
| (Ali, 2020) | Ali, H., Siddique, M., Pervez, M., Kumar, S., & Sami, W. (2020). Comparison of 5 fluorouracil and triamcinolone acetonide intralesional injection in the management of keloid. *Rawal Med J*, *45*(3), 549-53. |
| (Haurani, 2009) | Haurani, M. J., Foreman, K., Yang, J. J., & Siddiqui, A. (2009). 5-Fluorouracil treatment of problematic scars. *Plastic and reconstructive surgery*, *123*(1), 139-148. |
| (Ismail, 2020) | Ismail, S. A., Mohammed, N. H., Sotohy, M., & Abou-Taleb, D. A. (2021). Botulinum toxin type A versus 5-Fluorouracil in treatment of keloid. *Archives of Dermatological Research*, *313*, 549-556. |
| (Kabel, 2016) | Kabel, A. M., Sabry, H. H., Sorour, N. E., & Moharm, F. M. (2016). Comparative study between intralesional injection of bleomycin and 5-fluorouracil in the treatment of keloids and hypertrophic scars. *Journal of Dermatology & Dermatologic Surgery*, *20*(1), 32-38. |
| (Khare, 2012) | Khare, N., & Patil, S. B. (2012). A novel approach for management of ear keloids: results of excision combined with 5-fluorouracil injection. *Journal of Plastic, Reconstructive & Aesthetic Surgery*, *65*(11), e315-e317. |
| (Mohamad, 2022) | Ezzat Mohamad, N., Abd El Raheem, T. A., Mahmoud, R. H., & Osama Hamed, N. (2022). Evaluating serum level of thymidylate synthase in post burn keloid patients before and after intralesional injection of 5-fluorouracil. *Scars, Burns & Healing*, *8*, 20595131211049043. |
| (Monteiro, 2022) | Monteiro, R. C., Bhat, M. R., Martis, J., & Kamath, H. G. (2022). A comparative study of the efficacy of intralesional 5 fluorouracil vs combination of 5 fluorouracil with triamcinolone acetonide in keloids. *Indian Journal of Dermatology*, *67*(3), 211. |
| (Paltatzidou, 2017) | Paltatzidou, K., K. Xenos, A. Panagiotopoulos, E. Pouliou, E. Katsika‐Chatziolou, P. Stavropoulos, A. Katsambas, A. Stratigos, and C. Antoniou. "Localization of MMP‐9 in multinuclear giant cells in keloids after treatment with 5‐fluorouracil with or without combination of cryotherapy and cryotherapy alone." *Journal of the European Academy of Dermatology and Venereology* 31, no. 2 (2017): e121-e123. |
| (Rani, 2022) | Rani, T. U., Shanker, V. K., Vengareddy, S., Krishna, M., Thotli, R., Sushrutha, A., & Makarand, M. (2022). COMPARATIVE STUDY OF VARIOUS TOPICAL AND SURGICAL TREATMENT MODALITIES IN KELOID. *Int J Acad Med Pharm*, *4*(4), 449-457. |
| (Saha, 2012) | Saha, A. K., & Mukhopadhyay, M. (2012). A comparative clinical study on role of 5-flurouracil versus triamcinolone in the treatment of keloids. *Indian Journal of Surgery*, *74*, 326-329. |
| (Sharma, 2021) | Sharma, S., Vinay, K., & Bassi, R. (2021). Treatment of small keloids using intralesional 5-fluorouracil and triamcinolone acetonide versus intralesional bleomycin and triamcinolone acetonide. *The Journal of Clinical and Aesthetic Dermatology*, *14*(3), 17. |
| (Alhamzawi, 2021) | Alhamzawi, N. K. (2021). Efficacy of fractional carbon dioxide laser (FCO2) with intralesional 5-fluorouracil (5-FU) in the treatment of keloids. *Journal of Cutaneous and Aesthetic Surgery*, *14*(3), 323. |
| (Gupta, 2002) | Gupta, S., & Kalra, A. (2002). Efficacy and safety of intralesional 5-fluorouracil in the treatment of keloids. *Dermatology*, *204*(2), 130-132. |
| (Kontochristopoulos, 2005) | Kontochristopoulos, G., Stefanaki, C., Panagiotopoulos, A., Stefanaki, K., Argyrakos, T., Petridis, A., & Katsambas, A. (2005). Intralesional 5-fluorouracil in the treatment of keloids: an open clinical and histopathologic study. *Journal of the American Academy of Dermatology*, *52*(3), 474-479. |
| (Nanda, 2004) | Nanda, S., & Reddy, B. S. N. (2004). Intralesional 5‐fluorouracil as a treatment modality of keloids. *Dermatologic surgery*, *30*(1), 54-57. |
| (Reinholz, 2020) | Reinholz, M., Guertler, A., Schwaiger, H., Poetschke, J., & Gauglitz, G. G. (2020). Treatment of keloids using 5‐fluorouracil in combination with crystalline triamcinolone acetonide suspension: evaluating therapeutic effects by using non‐invasive objective measures. *Journal of the European Academy of Dermatology and Venereology*, *34*(10), 2436-2444. |
| (Wang, 2021) | Wang, H., Zhao, W., Xu, M., & An, L. (2021). Treatment of keloids by surgical excision combined with 5-fluorouracil and betamethasone injection. *Dermatologic Surgery*, *47*(5), 740-741. |
| (Wilson, 2013) | Wilson, A. M. (2013). Eradication of keloids: surgical excision followed by a single injection of intralesional 5-fluorouracil and botulinum toxin. *Canadian Journal of Plastic Surgery*, *21*(2), 87-91. |
| (Davison, 2009) | Davison, S. P., Dayan, J. H., Clemens, M. W., Sonni, S., Wang, A., & Crane, A. (2009). Efficacy of intralesional 5-fluorouracil and triamcinolone in the treatment of keloids. *Aesthetic surgery journal*, *29*(1), 40-46. |
| (Yuan, 2022) | Yuan, Chen, Xiaofeng Zhang, Yan Yan, Baoxi Wang, Zhenzhen Li, and Li Li. "Sequential therapy with intralesional injections and superficial X‐ray therapy in 96 keloids." *Journal of Cosmetic Dermatology* 21, no. 10 (2022): 5276-5278. |

**Supplemental Table S2.** Characteristics of studies with 5-fluorouracil monotherapy and non-monotherapy groups.

| **First author, publication year** | **Study Design** | **Patients; % Male** | **Mean age (range)** | **Study Quality (GRADE)** |
| --- | --- | --- | --- | --- |
| (Albalat, 2022) | RCT | 160; 31.9% | 32.25 (20-60) | High |
| (Ali, 2021) | RCT | 150; 52.7% | 25.94 (20-50) | High |
| (Ali, 2016) | RCT | 62; 38.7% | 31.76 (15-60) | High |
| (Asilian, 2012) | RCT | 69; 40% | 24.7 (NI) | High |
| (Chen, 2017) | RCT | 69; 42% | 26.7 (NI) | High |
| (Darougheh, 2007) | RCT | 47; 37.5% | NI (5-70) | High |
| (Deng, 2021) | RCT | 31; 38.7% | NI (19-48) | High |
| (Hatamipour, 2011) | RCT | 50; 40% | NI (22-45) | High |
| (Hietanen, 2020) | RCT | 49; 53.1% | 43.4 (18-81) | High |
| (Khalid, 2019) | RCT | 120; 42.6% | 29.4 (NI) | High |
| (Khalid, 2018) | RCT | 60; 26.7% | 31.8 (12-65) | High |
| (Li, 2022) | RCT | 60; 32.7% (of final sample) | 29.5 (NI) | High |
| (Manzoor, 2020) | RCT | 90; 51.1% | 28.2 (18+) | High |
| (Naseem, 2022) | RCT | 60; 43.3% | 32.9 (NI) | High |
| (Sadeghinia, 2011) | RCT | 40; NI | 44.2 (NI) | High |
| (Sagheer, 2016) | RCT | 60; 28.3% | 23.4 (13-42) | High |
| (Saleem, 2017) | RCT | 100; 57% | 31.6 (20-45) | High |
| (Sharma, 2012) | RCT | 28; 57.1% | NI (NI) | High |
| (Srivastava, 2018) | RCT | 60; 43.3% | 27.9 (18-56) | High |
| (Alamdari, 2018) | RCT | 33; 39.4% | NI (20-52) | High |
| (Ali, 2020) | Prospective comparative | 60; 46.7% | 35.97 (NI) | Moderate |
| (Haurani, 2009) | Prospective comparative | 35; 21.9% (of final sample) | NI (NI)  *median* = 25 (final sample) | Moderate |
| (Ismail, 2020) | Prospective comparative | 50; NI | 30.7 (15-55) | Moderate |
| (Kabel, 2016) | Prospective comparative | 120; NI | 31.23 (16-54) | Moderate |
| (Khare, 2012) | Prospective comparative | 60; 0% | NI (14-56) | Moderate |
| (Mohamad, 2022) | Prospective comparative | 40; NI | 28.5 (15-50) | Moderate |
| (Monteiro, 2022) | Prospective comparative | 30; 56.7% | NI (NI) | Moderate |
| (Paltatzidou, 2017) | Prospective comparative | 21; 66.7% | NI (NI) | Moderate |
| (Rani, 2022) | Prospective comparative | 80; 43.75% | NI (11-50) | Moderate |
| (Saha, 2012) | Prospective comparative | 50; NI | 33.83 (16-66) (final sample) | Moderate |
| (Sharma, 2021) | Prospective comparative | 40; NI | NI (18-60) | Moderate |
| (Alhamzawi, 2021) | Prospective non-comparative | 24; 58.3% | 24.25 (16-58) | Moderate |
| (Gupta, 2002) | Prospective non-comparative | 24; 50% | NI (NI) | Moderate |
| (Kontochristopoulos, 2005) | Prospective non-comparative | 20; 55% | NI (12-65) | Moderate |
| (Nanda, 2004) | Prospective non-comparative | 28; 42.9% | NI (11-55) | Moderate |
| (Reinholz, 2020) | Prospective non-comparative | 25; 76% | 28.8 (19-52) | Moderate |
| (Wang, 2021) | Prospective non-comparative | 21; 57.1% | NI (21-60) | Moderate |
| (Wilson, 2013) | Prospective non-comparative | 80; 37.5% | 24.7 (16-42) | Moderate |
| (Davison, 2009) | Retrospective comparative | 94; NI | NI (NI) | Moderate |
| (Yuan, 2022) | Retrospective non-comparative | 38; NI | NI (NI) *median =* 30 | Moderate |

*GRADE,* Grading of Recommendations, Assessment, Development, and Evaluation; *NI,* no information.

**Supplemental Table S3.** Anatomical location and number of keloids injected with 5-fluorouracil for included studies with both 5-fluorouracil monotherapy and non-monotherapy groups.

| **First author, publication year** | **Total Number of Keloids** | **Ear** | **Trunk** | **Limbs** | **Head and Neck** | **Other** |
| --- | --- | --- | --- | --- | --- | --- |
| (Alhamzawi, 2021) | 44 | 7 (15.9%) | 34 (77.3%) | 3 (6.8%) | N/A | N/A |
| (Ali, 2020) | 60 | 13 (21.7%) | 36 (60%) | 11 (18.3%) | N/A | N/A |
| (Deng, 2021) | 42 | N/A | 39 (92.9%) | 3 (7.1%) | N/A | N/A |
| (Khalid, 2018) | 30 | 30 (100%) | N/A | N/A | N/A | N/A |
| (Khare, 2012) | 28 | 28 (100%) | N/A | N/A | N/A | N/A |
| (Kontochristopoulos, 2005) | 20* | 1 (5%) | 14 (70%) | 5 (25%) | N/A | N/A |
| (Li, 2022) | 38 | N/A | 31 (81.6%) | 5 (13.2%) | 2 (5.3%) | N/A |
| (Nanda, 2004) | N/A | N/A | U/A (62.5%) | U/A (21.4%) | N/A | U/A (21.4%) |
| (Sagheer, 2016) | 60 | 2 (3.33%) | 53 (88.3%) | N/A | 5 (8.3%) | N/A |
| (Wilson, 2013) | 80 | 12 (15%) | 40 (50%) | 8 (10%) | 20 (25%) | N/A |
| (Yuan, 2022) | 96 | 1 (1%) | 62 (64.6%) | N/A | 32 (33.3%) | 1 (1%) |
| (Srivastava, 2018) | 40 | N/A | 30 (75%) | 7 (17.5%) | 3 (7.5%) | N/A |
| (Ismail, 2020) | 35 | N/A | 27 (77.1%) | 4 (11.43%) | 4 (11.43%) | N/A |
| (Haurani, 2009) | 32 | 22 (69%) | 4 (12.5%) | 1 (3%) | 5 (16%) | N/A |
| (Gupta, 2002) | 39 | N/A | 31 (79.5%) | 5 (12.8%) | 3 (7.7%) | N/A |

*N/A*, not applicable; *U/A*, information not available. Studies that did not report keloid location or that mentioned some locations in passing were not included.

* Only 1 keloid per patient was treated (total 20). However, some patients had more than 1 keloid. It was not stated which of these was treated. Trunk was chosen in 3 instances of patients with keloids on both the trunk and arm.

**Supplemental Table S4.** 5-fluorouracil treatment protocol of included studies with both 5-fluorouracil monotherapy and non-monotherapy groups.

| **First author, publication year** | **Study design; GRADE** | **5FU monotherapy protocol *(number of patients)*** | **Non-5FU monotherapy protocols *(number of patients)*** | **Follow-up** |
| --- | --- | --- | --- | --- |
|  |  |  |  |  |
| (Hietanen, 2020) | RCT; High | 50 mg/mL *(24)* | 20 mg/mL TAC + 10 mg/mL lidocaine *(25)* | q3-4weeks for 12 weeks; 26 weeks post |
| (Alamdari, 2018) | RCT; High | 50 mg/mL (0.2 mL/cm^2^) *(U/A)* | Cryotherapy *(U/A)*  40 mg/mL (0.2 mL/cm^2^) TAC *(U/A)* | q4weeks for 24 weeks; 26 weeks post |
| (Albalat, 2022) | RCT; High | 50 mg/mL (0.01–0.02 mL/cm) *(40)* | 40mg/mL (2 mg/cm) TAC *(40)*  2.5mg/mL Verapamil (0.5mg/cm) *(40)*  PRP + 3% calcium chloride *(40)* | q3weeks for 18 weeks; 6 weeks post |
| (Alhamzawi, 2021) | Prospective non-comparative; Moderate | N/A | 50 mg/mL (1 mL/cm^2^, max 3mL) 5-FU + fractional carbon dioxide laser *(24)* | q4weeks for 24 weeks; 52 weeks post |
| (Ali, 2020) | Prospective comparative; Moderate | 50 mg/mL *(30)* | 50mg/mL 5-FU + 40 mg/mL TAC *(30)* | 48h, weekly for 4 weeks, then q2weeks for 8 weeks, q4weeks for 12 weeks; 26 weeks post |
| (Ali, 2016) | RCT; High | N/A | 40 mg/mL (0.1 mL) TAC + 50 mg/mL (0.9 mL) 5-FU *(31)*  40mg/mL (0.25 mL) TAC *(31)* | weekly for 8 weeks; 4 weeks post |
| (Ali, 2021) | RCT; High | N/A | 10 mg/mL (0.2 mL) 5-FU + 10 mg/mL (0.25 mL) TAC *(75)*  10 mg/mL (0.25 mL) TAC *(75)* | weekly for 12 weeks; 2 weeks post |
| (Asilian, 2012) | RCT; High | N/A | A) 40 mg/mL (0.1 mL) TAC *(23)*  B) 40 mg/mL (0.1 mL) TAC + 50 mg/mL (0.9 mL) 5-FU *(23)*  C) 40 mg/mL (0.1 mL) TAC + 50 mg/mL (0.9 mL) 5-FU + irradiation by 585-nm pulsed-dye laser (5-7.5 J/cm²) *(23)* | A) weekly for 8 weeks; 4 weeks post  B) weekly for 8 weeks; 4 weeks post  C) weekly for 8 weeks; 12 weeks |
| (Chen, 2017) | RCT; High | N/A | A) 1 mL Diprospan (2 mg betamethasone disodium phosphate and 5 mg betamethasone dipropionate) *(23)*  B) 25 mg/mL (0.5 mL) 5-FU + 1 mL Diprospan *(23)*  C) 5-FU + Diprospan + irradiation by 1064 nm Nd:YAG laser (90-100 J/cm²) *(23)* | q4weeks for 12 weeks; 13 weeks post |
| (Darougheh, 2007) | RCT; High | N/A | A) 40 mg/mL (0.25 mL) TAC *(20)*  B) 40 mg/mL (0.1 mL) TAC + 50 mg/mL (0.9 mL) 5-FU *(20)* | weekly for 8 weeks; 12 weeks post |
| (Davison, 2009) | RCT; High | N/A | A) 5-FU + TAC mixture (75% 5-FU and 25% TAC with 0.1 mL of solution per cm of lesion) *(52 lesions)*  B) 5-FU + TAC mixture (75% 5-FU and 25% TAC with 0.1 mL of solution per cm of lesion), with excision *(24 lesions)*  C) TAC with excision *(26 lesions)* | A) q4weeks for an unknown amount of time; post 26 weeks-312 weeks  B) q2weeks for 6 weeks; post 26 weeks-312 weeks  C) q2weeks for 6 weeks; post 26 weeks-312 weeks |
| (Deng, 2021) | RCT; High | N/A | A) 0.2 mL/cm³ of 5-FU + TAC mixture (0.6 mL of 2.5% 5-FU + 5 mL of 1% TAC + 1 mL of 2% lidocaine) *(16)*  B) 0.2 mL/cm³ of 5-FU + TAC mixture and Strontium-90 brachytherapy (15-20 Gy) *(15)* | A) q3weeks for 9 weeks; 52 weeks post  B) q3weeks for 12 weeks; 52 weeks post |
| (Mohamad, 2022) | Prospective comparative; Moderate | 50 mg/mL (0.2–0.5 mg/cm^2^) *(20)* | Healthy controls *(20)* | weekly for 4-12 weeks; no post |
| (Hatamipour, 2011) | RCT; High | N/A | Perilesional surgical excision + topical silicone + 50 mg/mL (0.6-1 mL) 5-FU *(25)*  Perilesional surgical excision + topical silicone *(25)* | weekly for 3 weeks, q4weeks for 8 weeks; 24 and 52 weeks post |
| (Khalid, 2018) | RCT; High | N/A | A) Excision + 150 mg 5-FU (0.2 mL/cm²) + TAC (9:1 ratio) *(30)*  B) Excision + radiotherapy (10 Gy in 2 fractions) *(30)* | A) q4weeks for 8 weeks; post 26 weeks  B) post q4weeks for 24 weeks |
| (Khalid, 2019) | RCT; High | N/A | 10 mg TAC *(51)*  45 mg 5-FU + 4 mg TAC *(57)* | weekly for 8 weeks; post 4 weeks |
| (Khare, 2012) | Prospective comparative; Moderate | N/A | A) Excision + 50-150 mg 5-FU *(28)*  B) TAC *(24)* | A) 2 weeks after excision; post q4weeks for 24 weeks, then every 26 weeks  B) q2weeks; post q4weeks for 24 weeks, then every 26 weeks |
| (Kontochristopoulos, 2005) | Prospective non-comparative; Moderate | 50 mg/mL (0.2-0.4 mL/cm² ) *(20)* | N/A | weekly for 7 weeks |
| (Manzoor, 2020) | RCT; High | 50mg/mL *(30)* | 50 mg/mL 5-FU + 40 mg/mL TAC *(30)*  40mg/mL TAC *(30)* | q4weeks for 24 weeks; no post |
| (Reinholz, 2020) | Prospective non-comparative; Moderate | N/A | 50 mg/mL 5-FU + 40 mg/mL TAC in a 3:1 ratio *(25)* | q4weeks for 16 weeks; post 52 weeks |
| (Li, 2022) | RCT; High | N/A | A) Excision + 250 mg/mL (2 mL) 5-FU + 7 mg/mL (1 mL) betamethasone + 2 mg/mL lidocaine (1 mL) *(20)*  B) 250 mg/mL (2 mL) 5-FU + 7 mg/mL (1 mL) betamethasone + 2 mg/mL lidocaine (1 mL) *(20)*  C) Excision + radiotherapy (3.5-4 Gy per fraction x 3 fractions) *(20)* | A) post-operatively then q4weeks for 16 weeks; post 16 weeks  B) q4 weeks for 16 weeks; post 16 weeks  C) post 16 weeks |
| (Monteiro, 2022) | Prospective comparative; Moderate | 50 mg/mL *(15)* | 50 mg/mL 5-FU (0.1 mL) + 40 mg/mL TAC (0.1 mL) *(15)* | q2weeks for 4 weeks, q4weeks for 12 weeks; 26 weeks post |
| (Nanda, 2004) | Prospective non-comparative; Moderate | 50 mg/mL (0.5-2 mL) *(28)* | N/A | weekly for 12 weeks; 12 weeks post |
| (Naseem, 2022) | RCT; High | N/A | 50 mg/mL (0.9 mL) 5-FU + 0.1 mL TAC for each 1 cm region *(30)*  40 mg/mL TAC for each 1 cm region *(30)* | q4weeks for 24 weeks; no post |
| (Paltatzidou, 2017) | Prospective comparative; Moderate | A) 50 mg/cc (max 2 cc) *(7)* | B) 50 mg/cc 5-FU + cryotherapy *(7)*  C) Cryotherapy *(7)* | A) weekly for 6 weeks; no post  B) q4weeks for 24 weeks; no post  C) q4weeks for 24 weeks; no post |
| (Sadeghinia, 2011) | RCT; High | 50 mg/mL (1 mL) *(20)* | 0.5 mL of 40mg/mL of TAC *(20)* | q4weekly for 12 weeks; q8weekly post for 32 weeks |
| (Sagheer, 2016) | RCT; High | 50mg/mL *(30)* | 50mg/mL 5-FU (0.9 mL) + 40mg/mL TAC (0.1 mL) (30) | q4weekly for 24 weeks*;* 24 weeks post |
| (Rani, 2022) | Prospective comparative; Moderate | A) 50 mg/mL *(20)* | B) Cryotherapy + TAC (10mg/mL) *(20)*  C) TAC *(20)*  D) Excision + 5% imiquimod *(20)* | A-C): q3weekly till the lesion completely flattened and follow up for the period of 26 weeks(*during treatment)*  D): daily for 8 weeks  post : q4weekly for 24 weeks |
| (Saleem, 2017) | RCT; High | N/A | A) 50 mg/mL (0.9 mL) 5-FU + 40 mg/mL (0.1 mL) TAC for each 1 cm area *(50)*  B) 40 mg/mL (0.1 mL) TAC for each 1 cm area *(50)* | A) q4weeks for 12 weeks; post q4weeks for 12 weeks  B) q4weeks for 12 weeks; post q4weeks for 12 weeks |
| (Saha, 2012) | Prospective comparative; Moderate | 50 mg/mL (2 mL) *(20)* | 40 mg/ml TAC max: 100 mg/session (2 mL) *(24)* | weekly for 6 weeks; 52 weeks post or until recurrence noted |
| (Sharma, 2012) | RCT; High | 50 mg/mL *(25)* | 5-FU 50mg/mL (0.9 mL) + 40 mg/mL TAC (0.1 mL) *(25)* | weekly for 4 weeks, q8weeks for 4 weeks, q4weeks for 12 weeks; 52 weeks post |
| (Wang, 2021) | Prospective non-comparative; Moderate | N/A | Excision + 5-FU + betamethasone *(21)* | q3weeks for 12 weeks; post 70 weeks |
| (Wilson, 2013) | Prospective non-comparative; Moderate | N/A | Surgical excision + 5FU (max dose 500mg) + Botulinum toxin (140 IU) *(80)* | q4weeks for 104 weeks post |
| (Sharma, 2021) | Prospective comparative; Moderate | N/A | Group A: TAC + 5-FU (1:9) 0.1 mL *(30)*  Group B: TAC (40mg/mL) 0.1mL + bleomycin (1.5 IU/mL) 0.25 mL *(30)* | q3weeks (maximum 10 sessions); 24 weeks post |
| (Yuan, 2022) | Retrospective non-comparative; Moderate | N/A | A) Betamethasone + 5-FU (25 mg/mL) *(N/A)*  B) X-ray total dose of 16–20Gy in four fractions *(N/A)* | 1. q4weeks for an unknown amount of time 2. weekly or q2weeks for an unknown amount of time   post: mean of 31 weeks |
| (Srivastava, 2018) | RCT; High | 50 mg/mL (2 mL) *(20)* | TAC (40mg/mL) + 5FU (50mg/mL) in 1:9 *(20)*  TAC 40 mg/mL *(20)* | q3weeksfor 24 weeks or until the keloid resolved; 30 weeks post |
| (Kabel, 2016) | Prospective comparative; Moderate | 50 mg/mL (0.2–0.4 mL/cm²) *(30)* | 40 mg/ml (0.1 mL) TAC + 50mg/mL (0.9 mL) 5-FU for max 2mL per session (0.2–0.4 ml/cm^2^) *(30)*  1.5 IU/ml Bleomycin (0.5-1 ml/cm^2^) *(60)* | q4weeks for 12 weeks; 52 weeks post |
| (Ismail, 2020) | Prospective comparative; Moderate | A) 50 mg/mL (0.2–0.4 mL/cm^3^) *(25)* | 1. Botulinum toxin type A *(25)* | A): q4weeks for 6 weeks; q4weeks for 12 weeks post  B) weekly for 6 weeks; q4weeks for 12 weeks post |
| (Haurani, 2009) | Prospective comparative; Moderate | N/A | Surgical excision + 50 mg 5-FU *(32)* | q4weekly (max 10 sessions); 52 weeks post |
| (Gupta, 2002) | Prospective comparative; Moderate | 50-150 mg *(24)* | N/A | weekly for 16 weeks; 12-24 weeks post |

*GRADE,* Grading of Recommendations, Assessment, Development, and Evaluation; *RCT,* randomized controlled trial; *TAC*, triamcinolone acetonide; *U/A*, information not available; *5-FU,* 5-fluorouracil.

**Supplemental Table S5.** Outcomes reported on in the results section of the included papers with both 5-fluorouracil monotherapy and non-monotherapy groups.

| **First author, publication year** | **POSAS** | **VSS** | **Height** | **Length** | **Width** | **Volume** | **Size** | **Pruritus** | **Induration** | **Erythema** | **Overall investigator-assessed improvement** |
| --- | --- | --- | --- | --- | --- | --- | --- | --- | --- | --- | --- |
| (Hietanen, 2020) |  |  | ✓ |  |  |  |  |  |  |  |  |
| (Alamdari, 2018) |  |  | ✓ |  |  | ✓ | ✓ |  |  |  |  |
| (Albalat, 2022) | ✓ |  |  |  |  |  |  |  |  |  | ✓ |
| (Alhamzawi, 2021) |  | ✓ | ✓ |  |  |  |  | ✓ |  |  | ✓ |
| (Ali, 2020) |  |  | ✓ |  |  |  |  |  |  |  | ✓ |
| (Ali, 2016) |  |  |  |  |  |  | ✓ |  |  |  | ✓ |
| (Ali, 2021) |  |  |  | ✓ | ✓ |  | ✓ |  |  |  | ✓ |
| (Asilian, 2012) |  |  | ✓ | ✓ | ✓ |  |  | ✓ | ✓ | ✓ | ✓ |
| (Chen, 2017) |  |  |  |  |  |  |  | ✓ |  | ✓ | ✓ |
| (Darougheh, 2007) |  |  | ✓ | ✓ | ✓ |  |  | ✓ | ✓ | ✓ | ✓ |
| (Davison, 2009) |  |  |  |  |  |  | ✓ | ✓ |  |  |  |
| (Deng, 2021) |  | ✓ | ✓ |  | ✓ |  |  |  |  |  |  |
| (Mohamad, 2022) |  |  |  |  | ✓ | ✓ |  | ✓ |  | ✓ | ✓ |
| (Hatamipour, 2011) |  |  |  |  | ✓ |  | ✓ | ✓ |  |  | ✓ |
| (Khalid, 2018) |  |  |  |  |  |  |  |  |  |  | ✓ |
| (Khalid, 2019) |  |  | ✓ |  |  |  |  |  |  |  | ✓ |
| (Khare, 2012) |  |  |  |  |  |  |  |  |  |  | ✓ |
| (Kontochristopoulos, 2005) |  |  |  |  |  | ✓ |  | ✓ |  |  | ✓ |
| (Manzoor, 2020) |  |  | ✓ |  |  |  | ✓ |  |  |  | ✓ |
| (Reinholz, 2020) | ✓ |  | ✓ |  | ✓ | ✓ |  | ✓ |  |  |  |
| (Li, 2022) | ✓ | ✓ |  |  |  |  |  | ✓ |  |  |  |
| (Monteiro, 2022) |  |  |  | ✓ | ✓ |  | ✓ |  |  |  | ✓ |
| (Nanda, 2004) |  |  | ✓ |  |  |  | ✓ | ✓ | ✓ |  | ✓ |
| (Naseem, 2022) | ✓ |  |  |  |  |  |  |  |  |  |  |
| (Paltatzidou, 2017) |  |  |  |  |  |  |  |  |  |  |  |
| (Sadeghinia, 2011) |  |  | ✓ |  |  |  | ✓ |  |  |  | ✓ |
| (Sagheer, 2016) |  |  | ✓ |  |  |  | ✓ |  |  |  | ✓ |
| (Rani, 2022) |  |  | ✓ |  |  |  | ✓ | ✓ | ✓ |  | ✓ |
| (Saleem, 2017) |  | ✓ |  |  |  |  |  |  |  |  | ✓ |
| (Saha, 2012) |  |  | ✓ | ✓ | ✓ | ✓ |  | ✓ |  |  | ✓ |
| (Sharma, 2012) |  |  | ✓ |  |  |  |  | ✓ |  |  |  |
| (Wang, 2021) | ✓ |  |  |  | ✓ |  |  |  |  |  |  |
| (Wilson, 2013) |  |  |  |  |  |  |  |  |  |  |  |
| (Sharma, 2021) |  | ✓ | ✓ |  |  |  |  | ✓ |  |  | ✓ |
| (Yuan, 2022) |  |  |  |  |  |  |  |  |  |  | ✓ |
| (Srivastava, 2018) |  | ✓ | ✓ |  |  |  |  | ✓ |  |  | ✓ |
| (Kabel, 2016) |  | ✓ | ✓ |  |  |  |  |  |  |  |  |
| (Ismail, 2020) |  |  | ✓ |  |  |  |  | ✓ |  |  | ✓ |
| (Haurani, 2009) |  |  |  |  |  | ✓ |  |  |  |  |  |
| (Gupta, 2002) |  |  | ✓ |  |  |  |  |  |  |  | ✓ |

*POSAS,* patient and observer scar assessment scale; *VSS,* Vancouver scar scale.

**Supplemental Table S6.** Percent improvement changes from 5-fluorouracil monotherapy groups for the treatment of keloids.

| **First author, publication year** | **POSAS** | **VSS** | **Height*** | **Length** | **Width*** | **Volume** | **Size** | **Pruritus** | **Induration** | **Erythema** | **Overall investigator-assessed improvement** |
| --- | --- | --- | --- | --- | --- | --- | --- | --- | --- | --- | --- |
| (Hietanen, 2020) |  |  | NR |  |  |  |  |  |  |  |  |
| (Alamdari, 2018) |  |  | -58% |  |  | -61% | -32% (SA) |  |  |  |  |
| (Albalat, 2022) | -57% |  |  |  |  |  |  |  |  |  | *>50%:* 22 (55%) *(POSAS)* |
| (Ali, 2020) |  |  | -43% |  |  |  |  |  |  |  | *>50%:* 18 (60%) *(height)* |
| Mohamad, 2022) |  |  |  |  | NR | NR |  | NR |  | NR | *>0%:* 18 (90%) *(erythema, thickness, and pruritus)* |
| (Kontochristopoulos, 2005) |  |  |  |  |  | NR |  | NR |  |  | *>25%:* 19 (95%); *>50%:* 17 (85%) *(volume)* |
| (Manzoor, 2020) |  |  | NR |  |  |  | NR |  |  |  | *>25%:* 29 (97%); *>50%:* 22 (73%) *(height and size)* |
| (Monteiro, 2022) |  |  |  | NR | NR |  | NR |  |  |  | *>25%:* 11 (73%); *>50%:* 10 (67%) *(height)* |
| (Nanda, 2004) |  |  | NR |  |  |  | 70% of patients | 100% of patients | NR |  | *>25%:* 26 (93%); *>50%:* 22 (79%) *(symptoms, size, height, and induration)* |
| (Paltatzidou, 2017) |  |  |  |  |  |  |  |  |  |  |  |
| (Sadeghinia, 2011) |  |  | -~82% | NR | NR |  |  | -~100% | -~83% | -~78% | *>25%:* 20 (100%); *>50%:* 19 (95%) *(pruritus, surface, height, induration, and erythema)* |
| (Sagheer, 2016) |  |  | NR |  |  |  | NR |  |  |  | *>50%:* 10 (33%) *(flattening and size)* |
| (Rani, 2022) |  |  | NR |  |  |  | NR | NR | NR |  | *>25%:* 17 (85%); *>50%:* 12 (60%) *(symptoms, size, height, and induration)* |
| (Saha, 2012) |  |  | NR | NR | NR | NR |  | -80% |  |  | *>25%:* 17 (85%); *>50%:* 13 (65%) *(volume)* |
| (Sharma, 2012) |  |  | NR |  |  |  |  | -71% of patients |  |  | *>25%*: 22 (88%); *>50%:* 18 (72%) *(flattening)* |
| (Srivastava, 2018) |  | NR | -89% |  |  |  |  | -98% |  |  |  |
| (Kabel, 2016) |  | -54% | NR |  |  |  |  |  |  |  |  |
| (Ismail, 2020) |  |  | NR |  |  |  |  | -35% |  |  | *>25%:* 26 (74%); *>50%:* 17 (49%) *(height)* |
| (Gupta, 2002) |  |  | NR |  |  |  |  |  |  |  | *>25%:* 14 (58%); *>50%:* 20 (83%) *(height)* |

*Height also regarded as flattening; Width also regarded as thickness

*NR,* not reported or unable to be determined from graphs presented*; POSAS,* patient and observer scar assessment scale; *SA,* surface area; *VSS,* Vancouver scar scale.

Unless otherwise indicated, percent decreases were reported regarding the decrease in the dimension/measurement, rather than the number of patients.

**Supplemental Table S7.** Percent improvement changes from 5-fluorouracil combination therapy groups for the treatment of keloids.

| **First author, publication year** | **Therapy (5FU + __)** | **POSAS** | **VSS** | **Height** | **Length** | **Width** | **Volume** | **Size** | **Pruritus** | **Induration** | **Erythema** | **Investigator-assessed overall improvement** |
| --- | --- | --- | --- | --- | --- | --- | --- | --- | --- | --- | --- | --- |
| (Alhamzawi, 2021) | FCO2 Laser |  | -65% | -83% |  |  |  |  | -100% of patients |  |  | *>25%:* 22 (92%); *>50%:* 19 (79%) *(VSS)* |
| (Ali, 2020) | TAC |  |  | -73% |  |  |  |  |  |  |  | *>50%*: 26 (87%) *(height)* |
| (Ali, 2016) | TAC |  |  |  |  |  |  | NR |  |  |  | *>50%:* 12 (67%) *(size)* |
| (Ali, 2021) | TAC |  |  |  | NR | NR |  | -60% |  |  |  | *>75%:* 43 (57%) *(size)* |
| (Asilian, 2012) | A) TAC + PDL  B) TAC |  |  | A)-79%  B) -77% | NR | NR |  |  | NR | NR | A) severe -> mild  B) severe -> mild | A) *>25%:* 18 (90%); *>50%*: 14 (70%)  B) *>25%:* 20 (100%); *>50%*: 8 (40%)  *(height, length, width, pruritus, induration, and erythema)* |
| (Chen, 2017) | A) Diprospan  B) Diprospan + Nd: YAG |  |  |  |  |  |  |  | NR |  | NR | A*) >50%:* 11 (48%)  B*) >50%:* 16 (69%)  *(erythema, toughness, and pruritus)* |
| (Darougheh, 2007) | TAC |  |  | -64% | NR | NR |  |  | NR | NR | severe -> mild | *>25%:* 17 (100%) *>50%:* 7 (40%)  *(height, length, width, pruritus, induration, and erythema)* |
| (Davison, 2009) | A) TAC  B) TAC + excision |  |  |  |  |  |  | A*)* 81%  B) 92% | A + B: -93% of patients |  |  |  |
| (Deng, 2021) | A) TAC  B) TAC + Strontium-90 brachytherapy |  | NR | NR |  | A) -54%  B) NR |  |  |  |  |  |  |
| (Hatamipour, 2011) | Topical silicone + excision |  |  |  |  | NR |  | NR | NR |  |  | *Complete*: 18 (75%) *Partial*: 5 (21%)  *(size, thickness, texture, and symptoms)* |
| (Khalid, 2018) | TAC + excision |  |  |  |  |  |  |  |  |  |  | *No recurrence 6 months post*: 22 (73%) |
| (Khalid, 2019) | TAC |  |  | -70% |  |  |  |  |  |  |  | *>50%* 4 weeks post: 25 (78%) *(height)* |
| (Khare, 2012) | Excision |  |  |  |  |  |  |  |  |  |  |  |
| (Manzoor, 2020) | TAC |  |  | NR |  |  |  | NR |  |  |  | >25%: 30 (100%); >50%: 27 (90%) (*height and size)* |
| (Reinholz, 2020) | TAC | -52% |  | *-*59% |  | -44% | -53% |  | -57% |  |  |  |
| (Li, 2022) | A) Excision + betamethasone  B) betamethasone | A) -46%  B) -28% | A) -55%  B) -37% |  |  |  |  |  | A) -67%  B) -50% |  |  |  |
| (Monteiro, 2022) | TAC |  |  |  | NR |  |  | NR |  |  |  | *>25%:* 10 (67%); *>50%:* 10 (67%) *(height)* |
| (Naseem, 2022) | TAC | NR |  |  |  |  |  |  |  |  |  | *>70%:* 28 (93%) *(POSAS)* |
| (Paltatzidou, 2017) | Cryotherapy |  |  |  |  |  |  |  |  |  |  |  |
| (Sagheer, 2016) | TAC |  |  | NR |  |  |  | NR |  |  |  | >50%: 22 (73%) *(flattening and decrease in size)* |
| (Saleem, 2017) | TAC |  | -72% |  |  |  |  |  |  |  |  | *“Effective”*: 49 (98%) *(NR)* |
| (Sharma, 2012) | TAC |  |  | NR |  |  |  |  | -100% of patients |  |  | *>25%:* 25 (100%); *>50%:* 24 (96%) *(flattening)* |
| (Wang, 2021) | Excision + betamethasone | -54% |  |  |  | -63% |  |  |  |  |  |  |
| (Wilson, 2013) | Botulinum toxin |  |  |  |  |  |  |  |  |  |  |  |
| (Sharma, 2021) | TCA |  | -53% | NR |  |  |  |  | -100% |  |  | *>25%:* 28 (93%); *>50%:* 25 (83%)  *(flattening)* |
| (Yuan, 2022) | Betamethasone |  |  |  |  |  |  |  |  |  |  | >75%: 38 (100%) *(NR)* |
| (Srivastava, 2018) | TAC |  | NR | -84% |  |  |  |  | -93% |  |  |  |
| (Kabel, 2016) | TAC |  | -55*%* | NR |  |  |  |  |  |  |  |  |
| (Haurani, 2009) | Excision |  |  |  |  |  | NR |  |  |  |  |  |

*NR,* not reported or unable to be determined from graphs presented*; POSAS,* patient and observer scar assessment scale; *PDL,* pulsed dye laser; *SA,* surface area; *TAC,* triamcinolone acetonide; *VSS,* Vancouver scar scale; *5-FU,* 5-fluorouracil.

**Supplemental Table S8.** Percent improvement changes from other comparator groups for the treatment of keloids.

| **First author, publication year** | **Therapy** | **POSAS** | **VSS** | **Height** | **Length** | **Width** | **Volume** | **Size** | **Pruritus** | **Induration** | **Erythema** | **Overall investigator-assessed improvement** |
| --- | --- | --- | --- | --- | --- | --- | --- | --- | --- | --- | --- | --- |
| (Hietanen, 2020) | TAC |  |  | NR |  |  |  |  |  |  |  |  |
| (Alamdari, 2018) | A) TAC  B) Cryotherapy |  |  | A) -61%  B) -78% |  |  | A) -78%  B) -96% | A) -53% (SA)  B) -82% (SA) |  |  |  |  |
| (Albalat, 2022) | A) Verapamil  B) TAC  C) Platelet Rich Plasma | A) -68%  B) -60%  C) -61% |  |  |  |  |  |  |  |  |  | *>50%:*  A) 32 (80%)  B) 30 (75%)  C) 29 (72%)  *(POSAS)* |
| (Ali, 2016) | TAC |  |  |  |  |  |  | NR |  |  |  | *>50%:* 4 (21%) *(size)* |
| (Ali, 2021) | TAC |  |  |  | NR | NR |  | -49*%* |  |  |  | *>75%:* 27 (36%) *(size)* |
| (Asilian, 2012) | TAC |  |  | -50% | NR | NR |  |  | NR | NR | severe -> mild | >25%: 13 *(*65%*)*; *>50%:* 3 (15%) *(height, length, width, pruritus, induration, and erythema)* |
| (Chen, 2017) | Diprospan |  |  |  |  |  |  |  | NR |  | NR | *>50%:* 3 (12*%*) *(erythema, toughness, and pruritus)* |
| (Darougheh, 2007) | TAC |  |  | -37% | NR | NR |  |  | NR | NR | severe -> mild | *>25%:* 10 (65%); >50%*:* 2 (15%)  *(height, length, width, pruritus, induration, and erythema)* |
| (Davison, 2009) | Excision + TAC |  |  |  |  |  |  | -73% | NR |  |  |  |
| (Hatamipour, 2011) | Excision + topical silicone |  |  |  |  | NR |  | NR | NR |  |  | *Complete*: 10 (43%) *Partial*: 8 (35%)  *(size, thickness, texture, and symptoms)* |
| (Khalid, 2018) | Excision + radiotherapy |  |  |  |  |  |  |  |  |  |  | *No recurrence at 6 months post*: 13 (43%) |
| (Khalid, 2019) | TAC |  |  | -46% |  |  |  |  |  |  |  | *>50%* 4 weeks post: 15 (44%) *(height)* |
| (Khare, 2012) | TAC |  |  |  |  |  |  |  |  |  |  |  |
| (Manzoor, 2020) | TAC |  |  | NR |  |  |  | NR |  |  |  | >25%: 27 (90%); >50%: 21 (70%) (*height and size)* |
| (Li, 2022) | Excision + radiotherapy | -42% | -54% |  |  |  |  |  | -60% |  |  |  |
| (Naseem, 2022) | TAC | NR |  |  |  |  |  |  |  |  |  | *>70%:* 19 (73%) *(POSAS)* |
| (Paltatzidou, 2017) | Cryotherapy |  |  |  |  |  |  |  |  |  |  |  |
| (Sadeghinia, 2011) | TAC |  |  | -~61% | NR | NR |  |  | -~18% | -~41% | -~82% | >25%: 17 (85%); >50%: 10 (50%) *(pruritus, surface, height, induration, and erythema)* |
| (Rani, 2022) | A) TAC  B) Cryotherapy + TAC  C) Excision + topical imiquimod |  |  | NR |  |  |  | NR | NR | NR |  | A) >25%: 20 (100%); >50%: 18 (90%)  B) >25%: 20 (100%); >50%: 20 (100%)  C) >25%: 20 (100%); >50%: 20 (100%)  *(symptoms, size, height, and induration)* |
| (Saleem, 2017) | TAC |  | -51% |  |  |  |  |  |  |  |  | *“Effective”:* 31 (62%) *(NR)* |
| (Saha, 2012) | TAC |  |  | NR | NR | NR | NR |  | -79% |  |  | >25%: 22 (92%); >50%: 16 (67%) *(volume)* |
| (Sharma, 2021) | Bleomycin |  | -73% | NR |  |  |  |  | -100% |  |  | >25%: 28 (93%); *>50%:* 26 (86%)  *(flattening)* |
| (Srivastava, 2018) | TAC |  | *NR* | -64% |  |  |  |  | -89% |  |  |  |
| (Kabel, 2016) | Bleomycin |  | -73% | NR |  |  |  |  |  |  |  |  |
| (Ismail, 2020) | Intralesional botulinum toxin A |  |  | NR |  |  |  |  | -55% |  |  | >25%: 32 (94%); >50%: 27 (79%) *(height)* |

*NR,* not reported individually or unable to be determined from graphs presented*; POSAS,* patient and observer scar assessment scale; *SA,* surface area; *TAC,* triamcinolone acetonide; *VSS,* Vancouver scar scale; *5-FU,* 5-fluorouracil.

**Supplemental Table S9.** Recurrence rates from the included papers with both 5-fluorouracil monotherapy and non-monotherapy groups.

| **First author, publication year** | **5-FU Monotherapy** | **5-FU Combination therapy** | **Other Comparators** |
| --- | --- | --- | --- |
| (Hietanen, 2020) | 13 (54%) at 24 weeks |  | 10 (40%) at 24 weeks |
| (Alamdari, 2018) |  |  |  |
| (Albalat, 2022) | 2 (5%) at 6 weeks |  | Verapamil: 1 (2.5%) at 6 weeks  TAC: 0 (0%) at 6 weeks  Platelet rich plasma: 0 (0%) at 6 weeks |
| (Alhamzawi, 2021) |  | FCO2: 4 (21%) at 52 weeks |  |
| (Ali, 2020) | 0 (0%) at 24 weeks | TAC: 0 (0%) at 24 weeks |  |
| (Ali, 2016) |  |  |  |
| (Ali, 2021) |  |  |  |
| (Asilian, 2012) |  |  |  |
| (Chen, 2017) |  |  |  |
| (Darougheh, 2007) |  |  |  |
| (Davison, 2009) |  |  |  |
| (Deng, 2021) |  | TAC: 18 (86%) at 52 weeks  TAC + Strontium-90 brachytherapy: 8 (44%) at 52 weeks |  |
| (Mohamad, 2022) |  |  |  |
| (Hatamipour, 2011) |  | Excision + topical silicone: 1 (4%) at 48 weeks | Excision + topical silicone: 5 (22%) at 48 weeks |
| (Khalid, 2018) |  | Excision + TAC: 0 (0%) at 24 weeks | Excision + radiotherapy: 0 (0%) at 24 weeks |
| (Khalid, 2019) |  | TAC: 10 (18%) at 88 weeks | TAC: 20 (39%) at 88 weeks |
| (Khare, 2012) |  | Excision: 1 (4%) at 88 weeks | TAC: 7 (22%) at 88 weeks |
| (Kontochristopoulos, 2005) | 9 (47%) at 48 weeks |  |  |
| (Manzoor, 2020) |  |  |  |
| (Reinholz, 2020) |  |  |  |
| (Li, 2022) |  | Excision + betamethasone + lidocaine: 2 (11%) at 36 weeks  Betamethasone + lidocaine: 4 (20%) at 36 weeks | Excision + radiotherapy: 1 (6%) at 40 weeks |
| (Monteiro, 2022) | 0 (0%) at 24 weeks | TAC: 0 (0%) at 24 weeks |  |
| (Nanda, 2004) | 0 (0%) at 24 weeks |  |  |
| (Naseem, 2022) |  |  |  |
| (Paltatzidou, 2017) |  |  |  |
| (Sadeghinia, 2011) |  |  |  |
| (Sagheer, 2016) | 3 (10%) at 24 weeks | TAC: 0 (0%) at 24 weeks |  |
| (Rani, 2022) |  |  |  |
| (Saleem, 2017) |  | TAC: 0 (0%) at 12 weeks | TAC: 0 (0%) at 12 weeks |
| (Saha, 2012) | 6 (35%) at 24 weeks |  | TAC: 8 (36%) at 24 weeks |
| (Sharma, 2012) | 0 (0%) at 48 weeks | TAC: 0 (0%) at 48 weeks |  |
| (Wang, 2021) |  | Excision + betamethasone: 0 (0%) at 64 weeks |  |
| (Wilson, 2013) |  | Botulinum toxin: 3 (4%) at 26-104 weeks |  |
| (Sharma, 2021) |  | TAC: 5 (23%) at 24 weeks | Bleomycin: 0 (0%) at 24 weeks |
| (Yuan, 2022) |  | Betamethasone: 22 (23%) at 28 weeks |  |
| (Srivastava, 2018) | 0 (0%) at 30 weeks | TAC: 0 (0%) at 30 weeks | TAC: 0 (0%) at 30 weeks |
| (Kabel, 2016) | 12 (40%) at 48 weeks | TAC: 14 (47%) at 48 weeks | Bleomycin: 0 (0%) at 48 weeks |
| (Ismail, 2020) | 8 (31%) at 12 weeks |  | Intralesional botulinum toxin A: 2 (9%) at 12 weeks |
| (Haurani, 2009) |  | Excision: 6 (19%) at 52 weeks |  |
| (Gupta, 2002) | 0 (0%) at 12-24 weeks |  |  |

*TAC,* triamcinolone acetonide; *5-FU,* 5-fluorouracil.
