# Supplementary figures and images for: Intralesional 5-Fluorouracil for Keloids: A Systematic Review
Source: J Cutan Med Surg. 2024 May 28;28(4):381–6. doi: 10.1177/12034754241256346 (PMC11403916; doi:10.1177/12034754241256346)

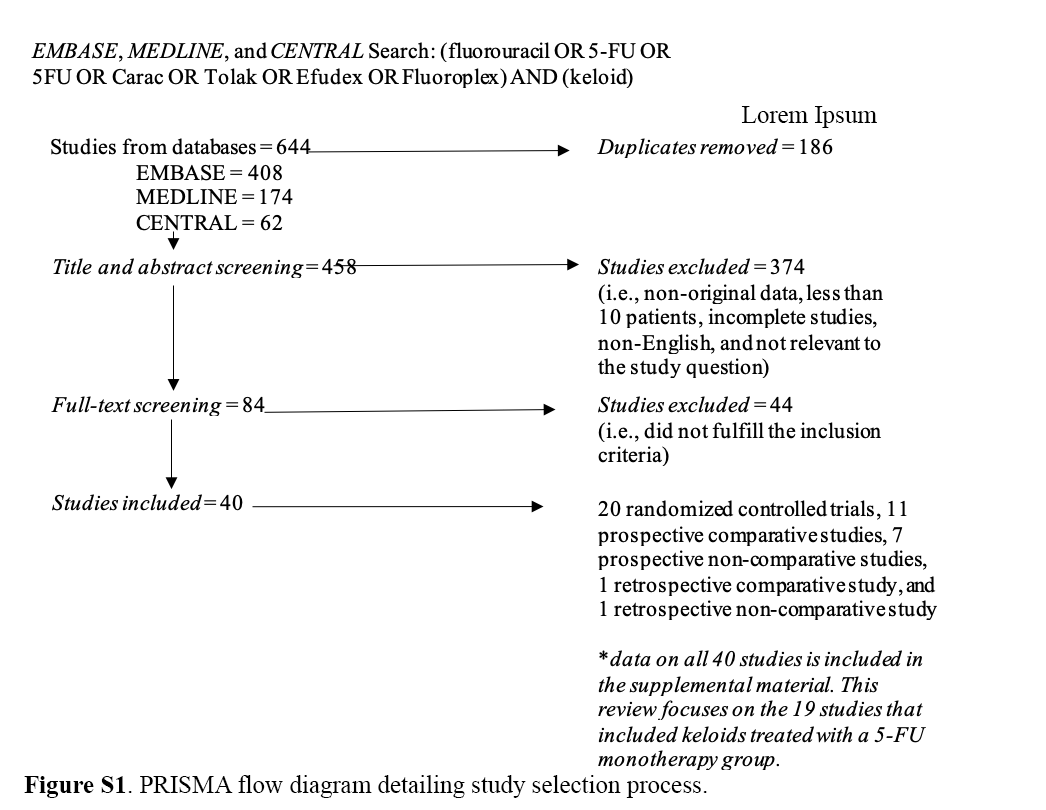

Supplement: sj-png-2-cms-10.1177_12034754241256346 – Supplemental material for Intralesional 5-Fluorouracil for Keloids: A Systematic Review [file sj-png-2-cms-10.1177_12034754241256346.png]
